# The contribution of reduction in malaria as a cause of rapid decline of under-five mortality: evidence from the Rufiji Health and Demographic Surveillance System (HDSS) in rural Tanzania

**DOI:** 10.1186/1475-2875-13-180

**Published:** 2014-05-10

**Authors:** Almamy M Kanté, Rose Nathan, Stéphane Helleringer, Mrema Sigilbert, Francis Levira, Honorati Masanja, Don de Savigny, Salim Abdulla, James F Phillips

**Affiliations:** 1 Mailman School of Public Health, Columbia University, 60 Haven Avenue, New York 10032, USA; 2 Ifakara Health Institute, PO Box 78373, Dar-Es-Salaam, Tanzania; 3 Swiss Tropical and Public Health Institute, University of Basel, Socinstrasse 57, CH-4002 Basel, Switzerland

**Keywords:** Under-five mortality, Malaria, Health and demographic surveillance system, Africa, Tanzania, Millennium development goal

## Abstract

**Background:**

Under-five mortality has been declining rapidly in a number of sub-Saharan African settings. Malaria-related mortality is known to be a major component of childhood causes of death and malaria remains a major focus of health interventions. The paper explored the contribution of malaria relative to other specific causes of under-five deaths to these trends.

**Methods:**

This paper uses longitudinal demographic surveillance data to examine trends and causes of death of under-five mortality in Rufiji, whose population has been followed for over nine years (1999–2007). Causes of death, determined by the verbal autopsy technique, are analysed with Arriaga’s decomposition method to assess the contribution of declining malaria-related mortality relative to other causes of death as explaining a rapid decline in overall childhood mortality.

**Results:**

Over the 1999–2007 period, under-five mortality rate in Rufiji declined by 54.3%, from 33.3 to 15.2 per 1,000 person-years. If this trend is sustained, Rufiji will be a locality that achieves MDG4 target. Although hypotrophy at birth remained the leading cause of death for neonates, malaria remains as the leading cause of death for post-neonates followed by pneumonia. However, declines in malaria death rates accounted for 49.9% of the observed under-five mortality decline while all perinatal causes accounted for only 19.9%.

**Conclusion:**

To achieve MDG 4 in malaria endemic settings, health programmes should continue efforts to reduce malaria mortality and more efforts are also needed to improve newborn survival.

## Background

The fourth Millennium Development Goal (MDG4) aims to achieve a two-thirds reduction in under-five mortality rate (U5MR) during 1990–2015 [1]. Between 1990 and 2010, the average U5MR in the world has fallen by only one-third from 89 to 60 deaths per 1,000 live births [2]. In 2011, 7.2 million children under-five died in the world with most of these deaths occurring in developing countries [3], of the regions of the world where achieving MDG4 has been most challenging, malaria has been a prominent cause of death. Yet, noteworthy progress has been registered in recent years. According to the Demographic and Health Surveys (DHS), the U5MR is declining in sub-Saharan Africa since 2000 after a decade of stagnation. Successive Tanzania DHS results have shown a decline of 40% of U5MR between 1992–1996 and 2006–2010 (from 137 to 81 per 1,000), with concomitant reduction in inequalities between age group, residence, maternal education and wealth quintile [4-7]. At the same time, U5MR declined faster in rural Tanzania than urban areas, from 85/1,000 to 60/1,000 against 73/1,000 to 63/1,000, respectively. Inequality in progress is nonetheless evident by age at death: post-neonatal mortality declined from 41/1,000 in 2001–2005 to 25/1,000 in 2006–2010 while neonatal mortality declined by only 30/1,000 to 26/1,000 in the corresponding period.

While malaria-related mortality is known to represent a major component of childhood causes of death in Tanzania, and malaria remains a major focus of health interventions, the possible contribution to malaria reduction in the Tanzania’s childhood mortality decline has yet to be investigated. This paper addresses this evidence gap. Improving scientific knowledge of childhood mortality trends is essential to guide efforts to prioritizing interventions and gauging the effectiveness of disease specific interventions [8-11]. In most developing countries however, there is a lack of precise data on demographic levels and trends because civil registration is incomplete. DHS in sub-Saharan Africa partially compensate for these gaps, but do not provide specific causes of death for children under-five years of age. Health and Demographic Surveillance systems (HDSS), a continuous monitoring of population in a delimited area, provide accurate information on mortality, fertility and migration trends [12]. The Rufiji HDSS (RHDSS), established in 1998 in a rural district of *Pwani* (Coast) Region, has collected data on causes of death by using the verbal autopsy (VA) study [13,14]. This paper analyses the contribution of malaria relative to other causes of death as components of the rapid U5MR decline in a rural district of coastal Tanzania.

## Methods

### Data collection

The RHDSS is located in Rufiji District about 178 km south of Dar es Salaam in eastern Tanzania between latitudes 7 °47′ and 8 °03′ South and longitudes 38 °62′ and 39 °17′ East. The RHDSS includes 38 of 94 villages of the district. Between 1999 and 2010, the monthly average temperature varied between 27.9 and 43.4°C and monthly rainfull between 0 and 420.7 mm [15].

In December 2007, the population under surveillance was 82,138 (47% of the district) and 15% were children under-five years old. The RHDSS area has a total of 18 health facilities (one hospital, two health centres and 15 dispensaries) and nearly 90% of the population lives within 5 km of a formal health facility [16].

**Figure 1:**
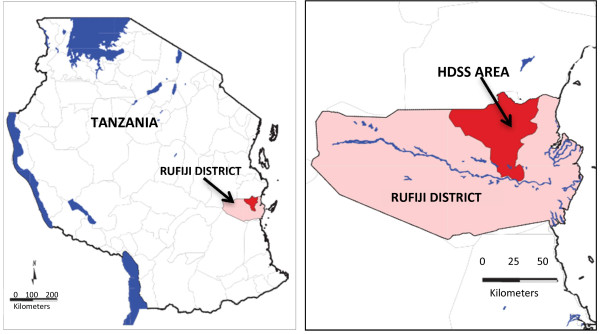
Map of the Rufiji Health and Demographic Surveillance System (RHDSS), Tanzania.

Launched in November 1998 with a baseline census of the surveillance population, the RHDSS has since been updated in 120 day intervals by revisits of interviewers who register the occurrence of all births, deaths, household in and out migrations, and marital status changes. To ensure completeness in reporting births and deaths of newborns, pregnancies are also documented and outcomes are solicited in subsequent household visits (Figure 1).

The causes of deaths were determined by the VA techniques. Deaths that occurred during interviewing rounds are recorded and specially-trained field researchers revisit the decedent’s home after a grieving period to conduct the interview. The VA forms are then independently reviewed by two physicians according to a list of causes of death based on the 10^th^ revision of the International Classification of Diseases [17]. A third physician codes the cause of death in case of discordant results. If there is disagreement among the three physicians, the death is coded as having an “undetermined” cause.

Based on these data, it is known that more than 95% of women attend at least at one antenatal visit, 65% deliver at a health facility and 80% of births are assisted by health professionals. Immunization coverage ranges from 85% for the Bacillus Calmette-Guerin (BCG; tuberculosis) to 66% for measles among children 12 to 23 months of age. Coverage rates for any nets were around 60% in 2010. The population covered by the RHDSS and in general the *Pwani* region, has better maternal health indicators, but worse child health indicators than circumstances prevailing elsewhere in rural Tanzania [7,13-16,18].

### Data analysis

The RHDSS data compiled over the January 1999 to December 2007 period, are used to calculate successive annual under age five mortality rates (per 1,000 person-years in observation, PYO). During this period, 2,691 childhood deaths were recorded and the main causes of deaths were reviewed using the VA method. About 20.8% of these deaths were reported as having undetermined causes and 5.7% were also not coded indicating the VA interviewer could not locate an appropriate respondent. Finally, 26.5% of under-five deaths were *unknown* causes.

While the frequency of unknown causes is high, evidence suggests that the age and sex composition of deceased children with known causes is similar to the corresponding composition of characteristics of deceased children with unknown causes for each year in which HDSS data were compiled. Chi-square tests are used to gauge percent differences of between known and unknown causes by demographic characteristic of deceases, season and period of death. Moreover, most of these characteristics exhibit similar patterns. For example, 74.0% of causes of death among boys were known compared to 73.0% among girls (p = 0.65). Also, 27.7% of deaths occurring during the raining season were unknow compared to 25.4% of deaths occuring during the dry season (p = 0.18). Maternal age data for children who died were also comparable (p > 0.05). Significant differences have however been noted for the age-group of children and the period of deaths. Twenty-three percent of neonatal death (less than one month of age) were unknown compared to 27.5% of postneonatal deaths (1–11 months) and 27.6% of post-infant deaths (12–59 months) (p = 0.05) (Table 1).

**Table 1:** Repartition of known and unknown under-five causes of deaths by characteristics of children, mothers, period and season of death in the Rufiji Health and Demographic Surveillance System (RHDSS), Tanzania, 1999–2007

**Cause of death**	**Sex**		**Age group of child (month)**			**Season of death**		**Period of death**		**Age group of mother (year)**		
	**Boy**	**Girl**	**< 1**	**1_11**	**12_59**	**Dry**	**Rain**	**1999-2002**	**2003-2007**	**< 20**	**20-30**	**>30**
Known	74.0	73.0	**76.8**	**72.5**	72.4	74.6	72.3	**76.6**	**70.2**	71.9	73.5	74.8
Unknown	26.0	27.0	**23.2**	**27.5**	27.6	25.4	27.7	**23.4**	**29.8**	28.1	26.5	25.2
Total	100.0	100.0	100.0	100.0	100.0	100.0	100.0	100.0	100.0	100.0	100.0	100.0
N	1,395	1,296	643	1,033	1,015	1,372	1,319	1,399	1,292	683	1,186	822

Significant at p < 0.05 level (in bold) (two-sided chi-square test).

Analysts have used contrasting approaches to accounting for unknown causes in the analysis of mortality. Black *et al.*[9] and Hope *et al.*[10] exclude unknown causes from their analyses, an approach that affects estimates of the mortality level and the pace of trends. Liu [19] and Narh-Bana *et al.*[20] retain such data in their analyses as an “unknown category” representing a residual class. Waltisperger and Meslé [21], Naghavi *et al.*[22] and Abdullah *et al.*[23] have taken into account the unknown causes by redistributing at random by age group and sex the unknown causes of death among the known causes in order to assure the comparability of series over the study period. This method avoids modification of the cause of death structure from known causes, preserving as a result, representativeness of known causes of deaths. This paper used proportional redistribution method of unknow causes into the known causes in order to minimize information loss.

The rate for each cause of death has been calculated, defined by the number of deaths due to the cause *x* occurring in a given period of time *t,* divided by the total number of person-years in observation (in per 1,000 PYO). Chi-square tests are also used to gauge differences of rates due to a cause *x* between two periods (*t*_*0*_*, t*_*1*_). The statistical significance is defined as a *p-value <0.05*.

To assess the proportionate contribution of each cause of death to the total change in life expectancy between *t*_*0*_ and *t*_*1*_, Chiang’s method has been used for constructing life tables to calculate life expectancy [24], then a decomposition technique proposed by Arriaga [25] has been employed, which permits examination of the contribution of each cause of death by periods. This method assumes that within the under-five age group, the contribution that a cause of death makes to a change in life expectancy between *t*_*0*_ and *t*_*1*_ is proportional to contribution that this cause makes to the change in the central mortality rate in that age group. The overall difference in the life expectancy between birth and age-five has been calculated between *t*_*0*_ and *t*_*1*_. Then the difference has been decomposed in proportional contribution of each cause of death into the overall decline of U5MR. Statistical analyses were executed using Stata 12 [26] and Microsoft Excel (2010).

This paper combined malaria and fever (acute febrile illness) (presumptive malaria) because during the early stage of the study (1999–2002), 27.4% of children deaths were attributed to fever without cough or respiratory distress, while that percent dropped to only 3.4% in the late stage (2003–2007). At the same time, the percent of children deaths attributed to malaria has increased from 6.7 to 25.9%.

### Ethical approval

Ethical approval was obtained from the Ministry of Health and Social Welfare, the National Medical Research Coordination Committee of National Institute for Medical Research and the Ifakara Health Institute (IHI)’s Institutional Review Board (IRB), Tanzania. Written informed consent was obtained from the patient’s guardian/parent/next of kin for the publication of this report and any accompanying images.

## Results

### Level and trends of under-five mortality rates

**Figure 2:**
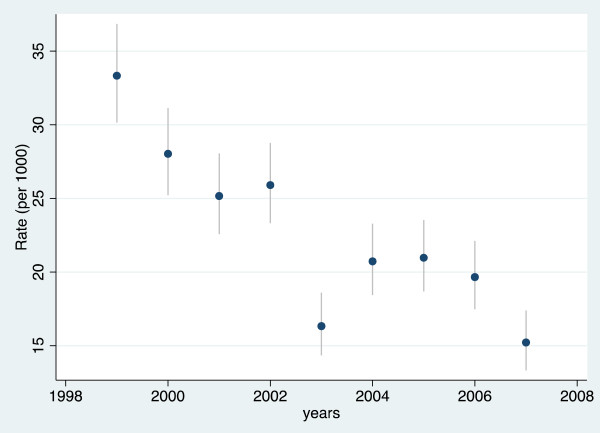
Under five mortality rates (per 1,000 person-years) trends in the Rufiji Health and Demographic Surveillance System (RHDSS), Tanzania, 1999–2007.

The U5MR significantly declined by 54.3% in nine years from 33.3 per 1,000 person-years in observation (PYO) in 1999 to 15.2/1,000 in 2007. To ensure the comparability of trends, two periods have been considered: 1999–2002 during which the mortality level was high at 28,1/1,000, then 2003–2007 during which mortality declined precipitously at 18.6/1,000 (*p < 0.0001*) (Figure 2).

### Cause of death of children under-five

The main causes of death of children under-five recorded in the RHDSS were malaria and fever (presumptive malaria), acute respiratory infections (pneumonia in particular), diarrhoeal diseases, malnutrition, birth injuries and asphyxia (BIA), prematurity and low birth weight (PLBW), congenital abnormalities and other perinatal causes (sepsis, neonatal tetanus).

Between 1999–2002 and 2003–2007, the overall under-five mortality rates have significantly declined by one-third (*p < 0.0001*). Presumptive malaria mortality rate declined significantly from 12.5 to 7.8/1,000 (*p < 0.0001*), pneumonia from 4.0 to 2.8/1,000 (*p < 0.001*) and also malnutrition, diarrhoeal diseases, congenital abnormalities and other perinatal causes (*p < 0.001*). However, anaemia has increased from 0.9 to 1.6/1,000 (*p < 0.001*) and BIA from 1.2 to 1.5/1,000 (*p = 0.15*) (Table 2).

**Table 2:** Under-five causes of death rates (per 1,000 person-year) by period in the Rufiji Health and Demographic Surveillance System (RHDSS), Tanzania, 1999-2007

**Cause of death**	**1999-2002**		**2003-2007**		***Overall***	
	**N**	**Death rates**	**N**	**Death rates**	***N***	**Death rates**
Malaria and fever	623	**12.5**	540	**7.8**	1163	9.8
Pneumonia	200	**4.0**	192	**2.8**	392	3.3
Diarrhoeal diseases	39	**0.8**	14	**0.2**	53	0.5
Anaemia	44	**0.9**	110	**1.6**	154	1.3
Malnutrition	86	**1.7**	63	**0.9**	149	1.3
Birth Injuries/Asphyxia	59	1.2	104	1.5	163	1.3
Prematurity/LBW	105	2.1	131	1.9	236	2.0
Congenital abnormalities	31	**0.6**	14	**0.2**	46	0.4
Other perinatal	105	**2.1**	37	**0.5**	142	1.2
Other	107	**2.2**	87	**1.3**	194	1.6
*Total*	**1399**	**28.1**	**1292**	**18.6**	2691	22.6
*Person-year*		49762.8		69419.3		119219.4

Rate = number of death due to cause x/person-year.

Bold = significant differences (p < 0.05) (Two-sided chi-square test).

Source: Database of Rufiji HDSS, 2012.

As a consequence of the improved survival situation from 1999–2002 and 2003–2007, the life expectancy between birth and age-five in Rufiji increased from 4.55 to 4.70 years corresponding to 1.73 months. Contribution of each cause of death to extending life expectancy is represented here by proportionate contributions into overall decrease of U5MR (Table 3).

**Table 3:** Contribution of under-five cause-specific death rates under-five in the Rufiji Health and Demographic Surveillance System (RHDSS), Tanzania, Arriaga’s method

**Cause of death**	**1999-2002 death rates**	**2003-2007 death rates**	**Difference in death rates**	**Proportionate contribution**	**Contribution**
Malaria/fever	12.5	7.8	4.7	49.9	0.8798
Pneumonia	4.0	2.8	1.2	13.1	0.2311
Diarrhoeal diseases	0.8	0.2	0.6	6.1	0.1080
Anaemia	0.9	1.6	−0.7	−7.2	−0.1275
Malnutrition	1.7	0.9	0.8	8.7	0.1538
Birth injuries/Asphyxia	1.2	1.5	−0.3	−3.3	−0.0587
Prematurity/LBW	2.1	1.9	0.2	2.2	0.0393
Congenital abnormalities	0.6	0.2	0.4	4.5	0.0788
Other perinatal	2.1	0.5	1.6	16.5	0.2905
Other causes	2.2	1.3	0.9	9.5	0.1670
**All causes**	**28.1**	**18.6**	**9.5**	**100.0**	**0.0002**

Source: Database of Rufiji HDSS, 2012.

The difference in U5MR is of 9.5/1,000 (28.1/1,000 against 18.6/1,000) between 1999–2002 and 2003–2007 comes from a combined death rates difference of presumptive malaria, pneumonia, diarrhoeal diseases, malnutrition, BIA, etc. The proportionate contribution by each cause is calculated by dividing the contribution of each cause by the change due to all causes. The major cause contributor to U5MR decline is presumptive malaria-related mortality, which explains half of the period differential in life expectancy. Other causes contributed to a relatively small proportion, perinatal causes (16.5%), pneumonia (13.1%), malnutrition (8.7%), diarrhoeal diseases (6.1%), congenital abnormalities (4.5%) and PLBW (2.2%). However, anaemia and BIA are higher over the 2003–2007 period, thus contributing to increasing mortality by 7.2 and 3.3% respectively.

**Figure 3:**
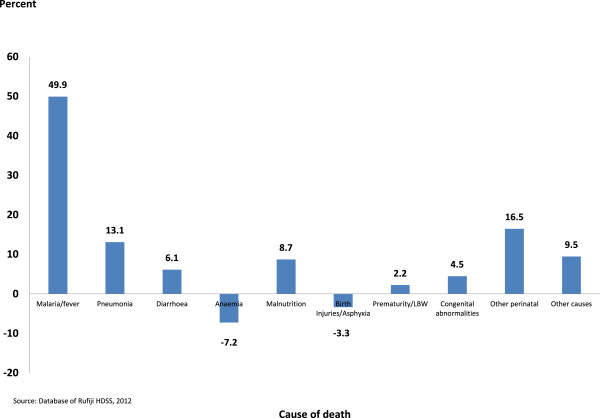
Proportionate contribution of under-five specific cause of death to the total change in life expectancy between birth and age five in the Rufiji Health and Demographic Surveillance System (RHDSS), Tanzania, 1999–2007.

In summary, between 1999–2002 and 2003–2007, presumptive malaria remained the major cause contributor to U5MR decline, 49.9%, while all perinatal causes contributed only 19.9%. While the presumptive malaria mortality rate significantly declined, it remains the leading cause of death followed by pneumonia. PLBW remained the leading cause of death for the neonates followed by the emergence of BIA (Figure 3).

## Discussion

Although important insights emerge from this analysis, results must be interpreted with caution owing to data limitations. Findings on cause of deaths trends in the RHDSS could be validated only (1) by supposing that the data collection protocol and diagnostic method of cause of death remained unchanged over the reference period and (2) by accepting the proportional redistribution method of unknown causes. The VA technique is widely used to ascribe childhood causes of death. This method is however known to be reliable for diagnosing only certain causes of deaths, such as measles, whooping cough, cholera, injuries and violence, while less so for delineating causes of associated with different febrile illnesses [27-30]. Caution must, therefore, be directed to ascribing to VA methods an ability to distinguish malaria from other causes of febrile illnesses that are common in the African context [31]. Although most of HDSS in sub-Saharan Africa are using VA methods with forms, procedures, and definitions that have not changed [32], VA data are compiled by field interviewers and medical VA readers. During the course of the project, different people rotated into these positions. Such subtle staffing changes may have introduced variation in the classification of causes of death over time. However, observers posit that such changes have a little impact on cause of death as diagnosed by physicians [33]. VA data also can be adjusted to allow for some of the misclassification biases [29] and for sensitivity and specificity in malaria mortality but this requires information on parasite prevalence also [32].

The proportion of unknown causes may also have an impact in the cause of deaths distribution. In 2002–2007, this proportion was however low in the RHDSS (19%) compared to Ifakara HDSS (31%), another HDSS in Tanzania [34]. The proportional redistribution method keeps unchanged cause of deaths trends over time. It allocates however a significant number to the most representative categories of causes of deaths. Thus, the proportionate contribution of presumptive malaria on U5MR decline would be at least 50% in RHDSS.

More recently, researchers have developed and applied standardized computer-based algorithm to assign causes of deaths using VA data. These probabilistic models have the advantage of being more internally consistent than physician generated causation estimates but mechanization of inference may lack essential subtlety and nuance [19,35-38]. These methods do not allocate specific cause for each death. They also produce undetermined causes, which should be included in the analysis [39]. This paper did not use such methods to compare with causes of deaths diagnosed by physicians. However, standardized computer-based algorithm methods can be useful to assess neonatal cause of deaths which remain more complicated to diagnose by physician [40].

The research legacy associated with the Rufiji study area may also have had an impact on results. The RHDSS, among other studies has been used as a platform of research on malaria. This focus on malaria and related pharmaceutical trials could influence the climate of diagnosis that physicians apply to causes of deaths. Due to their specialization, developed in the course of other studies, participating physicians may weight causes more heavily toward malaria than would be the case among physicians who lack this specialization [28,37].

The combining of malaria and fever (presumptive malaria) is related to the changes in the percent of children deaths attributed to fever or malaria between 1999–2002 and 2003–2007. That changes in the diagnose VA could be related, in part, to the concomitant introduction in most primary health care facilities of malaria rapid diagnostic tests (mRDTs) [41], the absence of any other new modality that would reduce mortality related to febrile illness [42] and to a large proportion of malaria deaths being properly recoded by the RHDSS.

These limitations, while important to note, are less likely to apply to inference related to long-term trend data than to simple cross-sectional studies. The RHDSS data, though flawed, are standardized over time. Changing compositions of estimated cause of death are more likely to arise from actual shifts in mortality risk than from shifts in any particular source of bias.

Over the past two decades, successive DHS in sub-Saharan Africa have documented a rapid decline of U5MR particularly in rural areas. However, these surveys do not provide the specific causes of deaths. Thus, HDSS in rural settings remain the main source providing accurate information on trends and causes of deaths in delimited area [31,43]. Results revealed that under-five mortality trends in the RHDSS were similar to the overall rural area in Tanzania [7]. If this trend is sustained, RHDSS and Tanzania as a whole [44] will be a locality that achieves the MDG4 target.

Second, three-quarters of the children under-five who died experienced one of only six causes (presumptive malaria, pneumonia, diarrheal diseases, malnutrition, PLBW and congenital abnormalities) as described for populations elsewhere in Africa [8-11]. Though all of these causes are in the process of decline, they remain the major killers of children in RHDSS. Nonetheless, their declining importance has dominated the process of U5MR decline in the RHDSS during the last decade.

Despite its high contribution to the U5MR decline, these results corroborate with the previous findings that malaria remains the leading cause of death among children in sub-Saharan Africa [45]. However, improvements in malaria prevention and care may explain why the decline in malaria-related mortality decline predominates as the cause contributing most to this progress. The successful introduction of preventive measures, particularly the insecticide-treated mosquito net [46-51] and more recent expansion of the availability of the mRDT [52,53] have had their intended impact. The introduction of mRDT has allowed immediate treatment of patients through efficient and low-cost drugs (Artemisinin-based combination therapy, ACT) [51,54]. Since March 2007, the ACT was introduced as first-line treatment for uncomplicated malaria and was provided free of charge through all fixed health facilities in Rufiji district [55]. Results from this study were also comparables to the Niakhar HDSS in Senegal [56] indicating that malaria-attributable mortality in under-5 children decreased from 13.5/1,000 during 1992–1999 to 2.2/1,000 in 2010 accelerating the achievement of MDG 4 in that rural area. That suggests that effective control of malaria can be sufficient to allow poor rural areas of Africa to meet MDG4.

Findings show that the contribution of anaemia to increase mortality goes against expected results regarding the contribution of malaria in that area of very high malarial transmission [57,58]. In rural Kenya, malaria and/or anaemia accounted for the greatest increases in U5MR in 2008–2009 due to stock-outs of essential anti-malarial drugs [59]. In the RHDSS, the slight increased of anaemia mortality rates between 2003 and 2007 could be related to other causes of deaths. Malaria alone is not the cause of anaemia and other possibilities include nutritional anaemia and hookworm infection [7].

Despite its contribution to mortality decrease, pneumonia remains one of most serious killers of young Tanzanians [7] as well as sub-Saharan African children elsewhere [9,60]. Mortality attributable to pneumonia has been reduced by third in the RHDSS over time probably due to reduction of the prevalence, the early diagnosis and the availability of treatment (antibiotics) with the successful introduction of the program known as the Integrated Management of Childhood Illness (IMCI) [61]. Most of the leading risk factors contributing to pneumonia incidence notably, lack of exclusive breastfeeding, malnutrition, indoor air pollution, low birth weight, crowding and lack of measles immunization [60] have been improved in the RHDSS over time.

Neonatal mortality remained high with only slight decline in the RHDSS and Tanzania in general [7,62,63]. PLBW and BIA remain the leading neonatal causes of deaths in the RHDSS as noted elsewhere in Africa [9,11].

## Conclusion

The results of this study suggest that several causes have contributed to rapid decline in U5MR in RHDSS. Presumptive malaria-related mortality was however the predominant contributor to the U5MR decline over the study period. Yet, despite this progress, presumptive malaria remains as a tragically high source of risk of mortality to young children. Thus, findings attest to the need (1) for renewed malaria control efforts and delivery of IMCI and (2) for improvement in the clinical management of complications of childbirth and preterm birth in commitment to strengthening maternal and newborn health to promote improved case of neonates. But the remaining challenge represented by malaria demonstrates that achieving MDG 4 requires continued attention to expanding efforts to reduce malaria related mortality.

## Abbreviations

ACT: Artemisinin-based combination therapy; BIA: Birth injuries and asphyxia; DHS: Demographic and Health Surveys; HDSS: Health and Demographic Surveillance System; IMCI: Integrated Management of Childhood Illness; MDG: Millennium Development Goal; PLBW: Prematurity and low birth weight; U5MR: Under-five mortality rates; RHDSS: Rufiji Health and Demographic Surveillance System; VA: Verbal autopsy.

## Competing interests

The authors declare that they have no competing interests.

## Authors’ contributions

AMK, SH and JFP conceived and designed the study. MS/FL prepared the dataset. AMK led the data analysis. AMK, JFP, RN, DS drafted the manuscript. All co-authors assisted the interpretation of results and made critical revisions of the manuscript. All authors read and approved the final manuscript.
